# Multi‐Omics Mendelian Randomization Identifies a DNA Methylation‐*ZDHHC20*‐Immune Axis Associated With Schizophrenia Risk

**DOI:** 10.1002/brb3.70722

**Published:** 2025-08-21

**Authors:** Qinghua Guo, Chao Chen, Yong Wang, Libo Guo, Shaomei Shang

**Affiliations:** ^1^ Outpatient Department Peking University Sixth Hospital; Peking University Institute of Mental Health; NHC Key Laboratory of Mental Health (Peking University); National Clinical Research Centerfor Mental Disorders (Peking University Sixth Hospital) Beijing China; ^2^ School of Nursing Peking University Beijing China; ^3^ Department of Nursing Peking University Sixth Hospital; Peking University Institute of Mental Health; NHC Key Laboratory of Mental Health (Peking University); National Clinical Research Centerfor Mental Disorders (Peking University Sixth Hospital) Beijing China

**Keywords:** Mendelian Randomization, DNA Methylation, ZDHHC20, Immune Axis

## Abstract

**Background:**

Schizophrenia is a severe psychiatric disorder with a complex etiology involving genetic and environmental factors. Despite its profound impact, the molecular mechanisms underlying schizophrenia remain elusive. Emerging evidence suggests that DNA methylation, palmitoylation, and immune cell activity play critical roles in its pathogenesis. This study employs a multi‐omics approach to investigate the causal relationships among these factors and schizophrenia.

**Methods:**

We utilized Mendelian randomization (MR), integrating expression, protein, and methylation quantitative trait loci (eQTL, pQTL, mQTL) to explore causal pathways in schizophrenia. Our approach involved (1) identifying palmitoylation‐related genes and assessing their causal effects on schizophrenia via two‐sample MR, validated by summary‐data‐based MR (SMR); (2) conducting mediation MR to examine upstream DNA methylation's role in gene regulation and its impact on schizophrenia; and (3) investigating downstream immune cell traits as mediators of gene effects on schizophrenia risk. Sensitivity analyses ensured robustness.

**Results:**

We found a significant causal association between increased ZDHHC20 expression, a palmitoyltransferase, and elevated schizophrenia risk (*p* < 0.05), confirmed by SMR. Upstream, DNA methylation at cg18095732 regulates *ZDHHC20*, mediating 59.31% of its effect on schizophrenia (*p* < 0.05). Downstream, CCR7 expression on naive CD8+ T cells mediates 33.35% of *ZDHHC20*’s influence on schizophrenia risk (*p* < 0.05).

**Conclusions:**

This study reveals a novel mechanistic axis in schizophrenia: DNA methylation‐ZDHHC20‐immune regulation, linking epigenetic modifications and palmitoylation to neuroimmune dysregulation. ZDHHC20 emerges as a potential therapeutic target, highlighting the value of multi‐omics in psychiatric disease research and suggesting avenues for targeted interventions addressing both neuronal and immune contributions.

**Abbreviations**
CCR7: Chemokine Receptor Type 7
CD8+T cells: Cluster of Differentiation 8 Positive T cells
CI: Confidence Interval
DALY: Disability‐Adjusted Life Year
eQTL: Expression Quantitative Trait Locus
GoDMC: Genetics of DNA Methylation Consortium
GWAS: Genome‐Wide Association Study
HEIDI: Heterogeneity In Dependent Instrument
IV: Instrumental Variable
IVW: Inverse Variance Weighted
LD: Linkage Disequilibrium
mQTL: Methylation Quantitative Trait Locus
MR: Mendelian Randomization
MR‐Egger: Mendelian Randomization Egger Regression
OR: Odds Ratio
pQTL: Protein Quantitative Trait Locus
SMR: Summary‐data‐based Mendelian Randomization
SNP: Single Nucleotide Polymorphism

## 1 Introduction

Schizophrenia is a chronic and severe neuropsychiatric disorder affecting approximately 80 million individuals globally, with a lifetime prevalence of 0.5–1% (Organization 2020). Data from 1990 to 2019 reveal a substantial increase in its burden, with the crude prevalence rising by over 65%, the incidence by 37.11%, and disability‐adjusted life years (DALYs) by 65% (Solmi et al. 2023). These figures underscore the escalating disease burden of schizophrenia.

Characterized by hallucinations, delusions, disordered thinking, and cognitive deficits, schizophrenia profoundly impacts affected individuals and society (Carpenter and Buchanan 1994, Tandon et al. 2013, Sabe et al. 2022). Typically emerging in late adolescence or early adulthood, the disorder varies in severity and progression. Despite its significant toll, the molecular mechanisms driving schizophrenia remain elusive. Genetic studies have pinpointed numerous associated loci, yet the intricate interplay of genetic and environmental factors complicates mechanistic understanding (Pantelis et al. 2014). Consequently, schizophrenia remains challenging to treat, with current therapies targeting symptoms rather than underlying causes.

Recent findings emphasize the need for deeper mechanistic insights, particularly highlighting the role of post‐translational modifications—such as palmitoylation—in neuronal function and disease progression. Palmitoylation, the covalent attachment of palmitic acid to proteins, modulates protein localization, stability, and interactions, influencing diverse cellular processes (Linder and Deschenes 2007, Li et al. 2023, Chamberlain and Shipston 2015). The ZDHHC family of acyltransferases, notably *ZDHHC20*, is a key regulator of neuronal palmitoylation (Chen et al. 2024). Given its involvement in synaptic plasticity and immune signaling, palmitoylation dysregulation may contribute to schizophrenia. Emerging evidence links palmitoylation‐related genes to the disorder's pathophysiology, though this connection warrants further exploration (Chen et al. 2024, Hornemann 2015, Liao et al. 2023, Buszka et al. 2023).

DNA methylation, a pivotal epigenetic modification, regulates gene expression and is implicated in psychiatric disorders, including schizophrenia (Jaffe et al. 2016, Starnawska and Demontis 2021). Methylation of cytosine residues in CpG dinucleotides alters gene activity without changing the DNA sequence, often via recruitment of repressive or activating complexes. Recent studies indicate that DNA methylation governs genes involved in neurotransmission, neurodevelopment, and immune function—processes potentially linked to schizophrenia (Shi et al. 2024, Xu et al. 2023). It is hypothesized that DNA methylation regulates palmitoylation genes, influencing cellular mechanisms underlying schizophrenia. As an upstream regulator, DNA methylation may control palmitoylation‐related gene expression, affecting immune cell activity and neuronal function, thus potentially bridging genetic risk factors and disease onset.

Immune dysfunction is increasingly recognized as a critical factor in schizophrenia's pathophysiology. Immune cells, including microglia and peripheral populations, contribute to neuroinflammation and synaptic plasticity in affected individuals (North et al. 2022, Du et al. 2024, Dietz et al. 2020). Emerging evidence suggests palmitoylation regulates immune cell function via protein trafficking and receptor signaling (Zheng et al. 2023, Shi et al. 2022), potentially influencing brain inflammation. Thus, palmitoylation genes may drive schizophrenia's development and progression through immune‐mediated effects. Elucidating the interplay between palmitoylation, immune function, and schizophrenia could reveal novel disease mechanisms and therapeutic targets.

In this study, we employ Mendelian randomization (MR) to investigate causal relationships between DNA methylation, palmitoylation, and schizophrenia. MR leverages genetic variants as instrumental variables to infer causality, minimizing confounding and reverse causation (Skrivankova et al. 2021, Sanderson et al. 2022).

We aim to examine DNA methylation's role in regulating *ZDHHC20* and its downstream effects on immune cell activity in schizophrenia. The study comprises three stages: first, using MR with eQTL and pQTL data to identify palmitoylation hotspot genes, validated by summary‐data‐based MR (SMR) analysis; second, exploring DNA methylation's mediating role in gene regulation and schizophrenia to uncover upstream mechanisms; and third, assessing downstream effects on immune cells and schizophrenia via mediation MR analysis. Specifically, we will investigate how DNA methylation in genes like ZDHHC20 influences immune‐mediated processes leading to schizophrenia onset. This research seeks to provide fresh insights into schizophrenia's molecular basis and identify new therapeutic avenues.

## 2 Methods

### 2.1 Study Design

**FIGURE 1:**
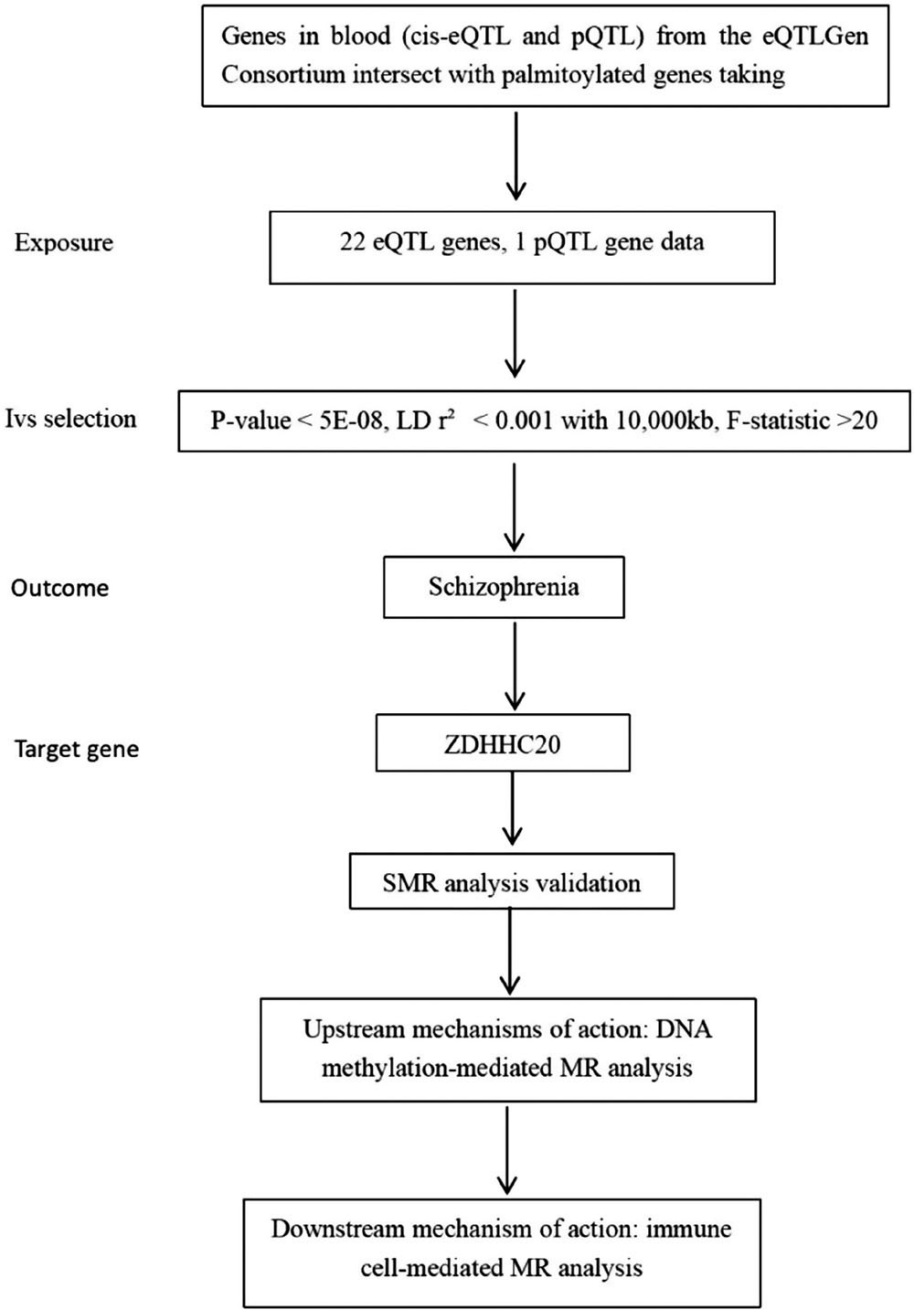
The flowchart of our study design. A three‐step MR framework was implemented to explore causal relationships between palmitoylation genes, DNA methylation, immune cells, and schizophrenia. The pipeline includes MR and SMR for gene selection, upstream methylation mediation, and downstream immune cell mediation.

The overall study design is depicted in Figure 1. In the first stage, we conducted a two‐sample MR analysis, intersecting genes from the deCODE GENETICS report (cis‐eQTL and pQTL) with those implicated in palmitoylation research (Li et al. 2023, Chamberlain and Shipston 2015, Chen et al. 2024). These intersecting gene sets served as exposures, with schizophrenia genome‐wide association study (GWAS) data as the outcome, to explore the causal relationship between palmitoylation‐related gene expression and schizophrenia susceptibility and progression. We selected suitable single nucleotide polymorphisms (SNPs) as instrumental variables (IVs) based on strict inclusion and exclusion criteria and performed sensitivity analyses to ensure MR analysis quality. For palmitoylation genes showing significant MR results, we conducted summary‐data‐based Mendelian randomization (SMR) analysis to validate our hypothesis. In the second stage, we used mediation MR analysis to investigate upstream mechanisms of DNA methylation in gene regulation and its association with schizophrenia. In the third stage, we explored downstream mechanisms by which target genes influence immune cells and affect schizophrenia, again using mediation MR analysis. Finally, we employed mediation MR to further examine the role of DNA methylation in palmitoylation gene regulation of immune cells and its potential contribution to schizophrenia development.

### 2.2 Exposure Data

In our MR analysis, we utilized multiple large‐scale genetic datasets as exposure data sources to investigate causal relationships between palmitoylation genes, DNA methylation, immune cell activity, and schizophrenia. These datasets provided IVs for the three study stages, assessing the effects of palmitoylation‐related gene expression, DNA methylation levels, and immune cell traits.

#### 2.2.1 Palmitoylation‐Related Gene Expression and Protein Levels

In the first stage, we focused on genes involved in palmitoylation, a reversible post‐translational modification mediated by ZDHHC family palmitoyltransferases affecting various substrates. We compiled a comprehensive list of palmitoylation‐related genes, including 23 human *ZDHHC* enzymes (e.g., *ZDHHC20*) and over 100 reported substrates (e.g., *SNAP25*, PSD‐95, and *RAS* family members) (Li et al. 2023, Chamberlain and Shipston 2015). These genes were cross‐referenced with deCODE GENETICS data to identify those with available cis‐expression quantitative trait loci (cis‐eQTL) and protein quantitative trait loci (pQTL). SNPs significantly associated with gene expression or protein levels (*p* < 5 × 10^−8^) were selected as IVs for two‐sample MR analysis, using schizophrenia GWAS summary statistics as the outcome. To ensure IV independence, we performed linkage disequilibrium (LD) clumping (*r^2^* < 0.001), minimizing potential bias in MR estimates.

#### 2.2.2 DNA Methylation Data

In the second stage, we examined the upstream regulatory role of DNA methylation in modulating palmitoylation‐related gene expression and its contribution to schizophrenia susceptibility. We utilized methylation quantitative trait loci (mQTL) data from the Genetics of DNA Methylation Consortium (GoDMC), a meta‐analysis resource providing genetic variants associated with DNA methylation levels at CpG sites in whole blood (http://mqtldb.godmc.org.uk/downloads). We targeted CpG sites within or near palmitoylation‐related genes identified in the first stage (e.g., *ZDHHC20*). mQTLs associated with CpG sites in promoter regions or gene bodies (*p* < 5 × 10^−8^) were selected as IVs and subjected to LD clumping (*r^2^* < 0.001) to ensure independence, enabling assessment of DNA methylation's causal impact on gene expression and schizophrenia risk in mediation MR analysis.

#### 2.2.3 Immune Cell Traits

In the third stage, we investigated downstream effects of palmitoylation‐related gene expression on immune cell activity and its mediating role in schizophrenia risk. We obtained GWAS summary statistics for immune cell traits from large‐scale public studies, associating genetic variants with phenotypes such as cell counts, surface marker expression, and functional assays in population cohorts (Orrù et al. 2020). We prioritized traits relevant to neuroinflammation and schizophrenia, including microglial activation markers, T‐cell subsets, and cytokine levels. SNPs associated with these traits (*p* < 5 × 10^−8^) were included in mediation MR analysis, with LD clumping (*r^2^* < 0.001) ensuring IV independence, allowing evaluation of whether immune cell activity mediates the relationship between palmitoylation genes and schizophrenia.

All genetic datasets were harmonized for consistent allele coding and effect direction. To reduce bias, only SNPs with a minor allele frequency > 1% and no significant LD (*r^2^* < 0.001) were included as IVs, supporting robust causal inference across the multi‐stage design.

### 2.3 Instrumental Variables (IVs) Selection

To ensure result reliability, MR analysis must satisfy three assumptions: (1) IVs are strongly associated with the exposure; (2) IVs are independent of confounders; and (3) IVs affect the outcome only via the exposure (no horizontal pleiotropy). We implemented a rigorous IV selection process:

Step 1: SNP screening

SNPs from cis‐eQTL data were selected with P‐values below the genome‐wide significance threshold (5.0 × 10^−8^), ensuring strong association with the exposure.

Step 2: Ensuring SNP independence

LD clumping (*r^2^* < 0.001, 10,000 kb window) was performed using 1000 Genomes European population data (Statistical methods for cis‐Mendelian randomization with two‐sample summary‐level data), ensuring IV independence.

Step 3: Excluding incompatible SNPs

Incompatible SNPs (e.g., A/G vs. A/C) were removed. For palindromic SNPs, positive strand alleles were inferred using allele frequency; those without frequency data were excluded.

Step 4: Assessing instrument strength

*F*‐statistics were calculated to evaluate IV strength and avoid weak instrument bias (Pierce et al. 2011):

$$F=\frac{R^{2}\times \left(n-1-K\right)}{K\times (1-R^{2})}$$

Where *R^2^*

$$\begin{array}{ccc} & & R^{2}\\ & & \;=\frac{2\times EAF\times \left(1-EAF\right)\times \beta^{2}}{2\times EAF\times \left(1-EAF\right)\times \beta^{2}+2\times EAF\times \left(1-EAF\right)\times N\times SE^{2}} \end{array}$$

SNPs with *F* < 20 were excluded.

This process ensured robust IVs meeting MR assumptions.

### 2.4 Outcome Data

Schizophrenia susceptibility GWAS data were sourced from FinnGen Data R11, including 6933 cases and 439,144 controls (https://storage.googleapis.com/finngen‐public‐data‐r11/summary_stats/finngen_R11_F5_SCHZPHR.gz).

### 2.5 Validation of MR Analysis and SMR Analysis of Palmitoylation Genes and Schizophrenia

In the first stage, MR analysis employed five methods: inverse variance weighted (IVW), weighted median, MR‐Egger, weighted mode, and simple mode (Burgess and Thompson 2017, Hartwig et al. 2017). IVW results were primarily reported due to superior performance (Bowden et al. 2016), with others as supplements. Causal significance was inferred when IVW *p* < 0.05 and effect directions aligned with MR‐Egger. Results were expressed as odds ratios (OR) with 95% confidence intervals (CI), analyzed using the “TwoSampleMR” R package (v0.5.6, R v4.3.1). Sensitivity analyses included Cochrane's *Q* test for heterogeneity (*p* < 0.05 indicating heterogeneity) and MR‐Egger for pleiotropy (intercept p < 0.05 suggesting pleiotropy) (Hemani et al. 2018). SMR analysis, using SMR software (v1.3.1), and HEIDI tests distinguished pleiotropy from linkage (Zhu et al. 2016), with SMR p < 0.05 and HEIDI p > 0.05 indicating shared genetic variation.

### 2.6 DNA Methylation MR Mediator Analysis

In the second stage, two‐step mediation MR assessed DNA methylation's role in regulating palmitoylation gene expression and schizophrenia susceptibility. Total effects were decomposed into indirect (mediated) and direct effects, estimating DNA methylation effects on schizophrenia (β), on palmitoylation genes (β₁), and palmitoylation genes on schizophrenia (β_2_). Mediation effect (β₁ × β_2_), proportion mediated ([β₁ × β_2_]/β), and direct effect were calculated (Burgess et al. 2019). Effects were retained if directionally consistent: positive β required β₁ and β_2_ to be both positive or both negative; negative β required opposite signs.

### 2.7 MR‐Mediated Analysis of Immune Cells

In the third stage, two‐step mediation MR evaluated immune cell activity's mediating role in palmitoylation gene effects on schizophrenia. Effects of palmitoylation genes on schizophrenia (β), on immune cells (β₁), and of immune cells on schizophrenia (β_2_) were estimated, with mediation effect (β₁ × β_2_), proportion mediated, and direct effect calculated (Burgess et al. 2019). Consistency rules mirrored those in 2.6.

### 2.8 MR Sensitivity Analysis

Sensitivity analyses used MR‐Egger, leave‐one‐out, and MR‐PRESSO tests to assess robustness (Burgess et al. 2017). Cochrane's *Q* tested heterogeneity (*p* < 0.05), MR‐Egger detected pleiotropy (*p* < 0.05), and MR‐PRESSO corrected horizontal pleiotropy.

## 3 Results

### 3.1 Palmitoylation Gene Selection

**FIGURE 2:**
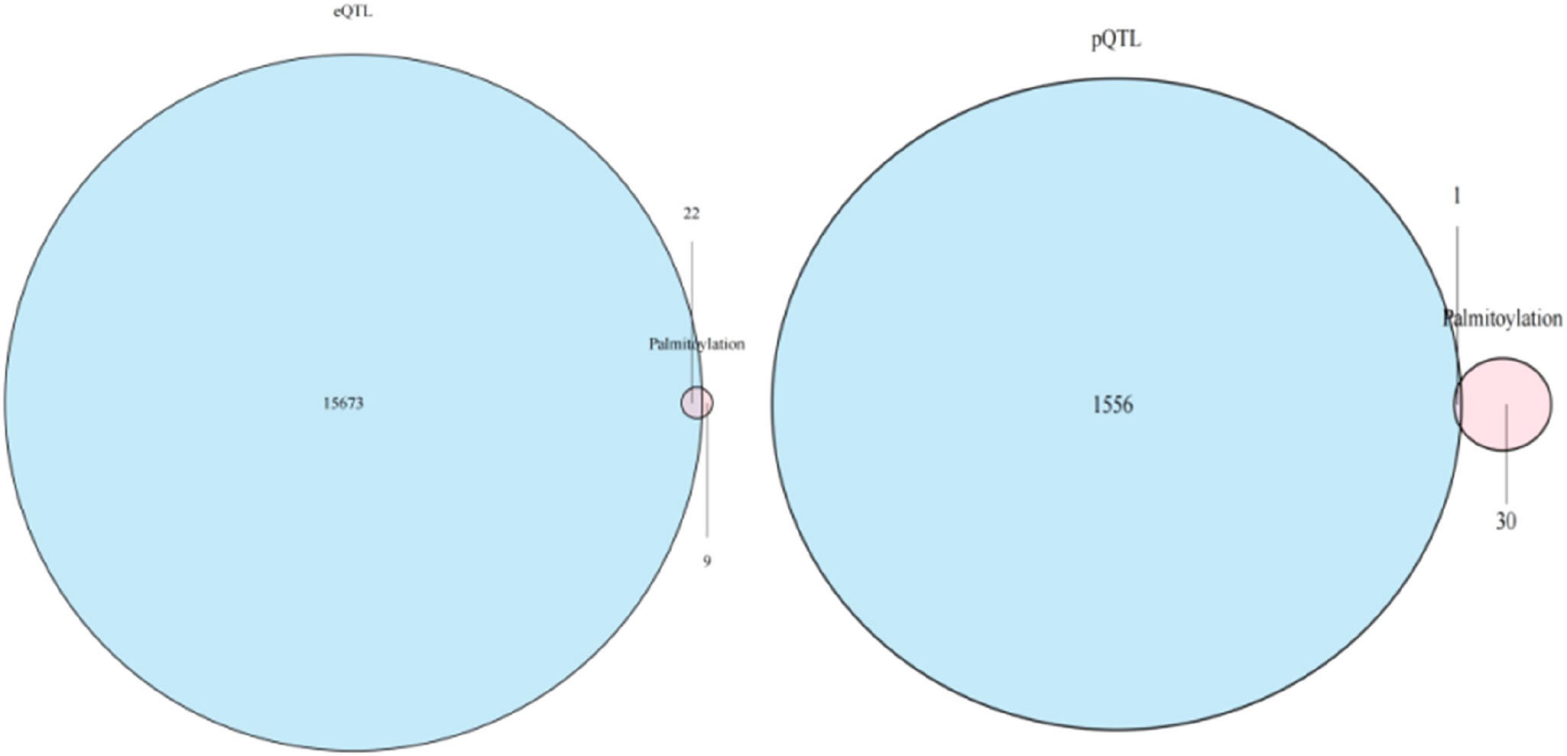
Palmitoylation gene selection by eQTL and pQTL. Palmitoylation‐related genes were cross‐referenced with eQTL and pQTL datasets, resulting in 22 eQTL genes and 1 pQTL gene for further MR analysis. *ZDHHC20* was highlighted as a central gene for schizophrenia susceptibility.

We integrated palmitoylation‐related genes with expression quantitative trait loci (eQTL) and protein quantitative trait loci (pQTL) data derived from transcriptomic and proteomic datasets of the Icelandic population. This analysis identified 22 eQTL genes and 1 pQTL gene associated with palmitoylation, as illustrated in **Figure** **2**. Further details are available in Supplementary Table S2.

### 3.2 MR Analysis and SMR Validation

For MR analysis, we selected 459 single nucleotide polymorphisms (SNPs) as IVs for 22 palmitoylation‐related eQTL genes and 13 SNPs for 1 pQTL gene, based on predefined IV criteria. All IVs exhibited F‐statistics greater than 20, confirming the absence of weak instrument bias. Comprehensive IV details are provided in Supplementary Table S3.

**FIGURE 3:**
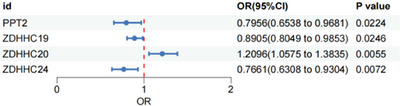
Significant MR results between palmitoylated gene expression and susceptibility to schizophrenia. MR revealed a significant causal link between ZDHHC20 expression and schizophrenia risk. SMR analysis validated this finding, identifying *ZDHHC20* as a novel risk gene.

Using eQTL data in MR analysis (primarily the inverse variance weighted [IVW] method), we detected significant associations between the expression of four palmitoylation genes—*PPT2*, *ZDHHC19*, *ZDHHC20*, and *ZDHHC24*—and schizophrenia susceptibility (*p* < 0.05), as shown in **Figure** **3** (see Supplementary Table S3 for details). No significant associations were observed with pQTL data. Cochran's *Q* test indicated no heterogeneity among the IVs for these genes, and MR‐Egger regression confirmed the absence of pleiotropy (Supplementary Table S4).

Additionally, the cis‐eQTL for *ZDHHC20* satisfied both the summary‐data‐based Mendelian randomization (SMR) test (p < 0.05) and the heterogeneity in dependent instruments (HEIDI) test (*p* > 0.05) (Supplementary Table S5). These results highlight *ZDHHC20* as a promising palmitoylation‐related candidate gene for schizophrenia.

### 3.3 DNA Methylation‐Mediated MR Analysis

**FIGURE 4:**
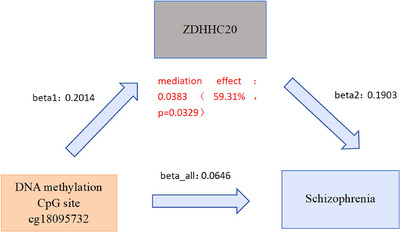
*ZDHHC20* palmitoylation gene mediates DNA methylation CpG locus cg18095732 in schizophrenia. Two‐step MR identified that DNA methylation at CpG site cg18095732 is associated with the regulation of *ZDHHC20* expression, which in turn is associated with schizophrenia risk. The estimated mediation proportion via *ZDHHC20* was 59.31%, suggesting a potential upstream epigenetic regulatory mechanism.

To investigate upstream mechanisms, we conducted MR analysis to assess how DNA methylation at CpG sites influences schizophrenia via the *ZDHHC20* palmitoylation gene. Using a two‐step MR approach with directional filtering of regression coefficients (β, β₁, and β_2_), we identified a potential pathway linking DNA methylation CpG sites, ZDHHC20, and schizophrenia (Supplementary Tables S6, S7, and S8). After calculating the mediation effect, we found that ZDHHC20 mediates the impact of the DNA methylation CpG site cg18095732 on schizophrenia, with a mediation effect of 0.0383, representing 59.31% of the total effect (**Figure** **4**; details in Supplementary Table S9).

### 3.4 Immune Cell‐Mediated MR Analysis

**FIGURE 5:**
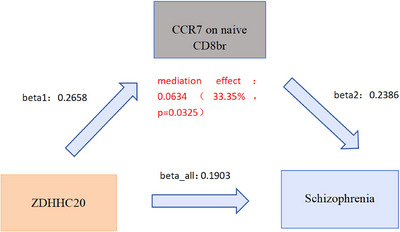
CCR7 on naive CD8br mediates the effect of the *ZDHHC20* palmitoylation gene on schizophrenia. Mediation Mendelian randomization analysis suggests that the association between *ZDHHC20* expression and schizophrenia risk may be partly mediated through CCR7 expression on naive CD8⁺ T cells, with a mediation proportion of 33.35%. These findings support a possible immune‐related downstream mechanism.

We further explored the downstream mechanism by which ZDHHC20 affects schizophrenia through immune cells, employing MR analysis. Using a two‐step MR method with directional filtering of β, β₁, and β_2_, we delineated a pathway connecting *ZDHHC20*, immune cells, and schizophrenia (Supplementary Tables S10, S11, S12, S13, and S14). Our analysis revealed that CCR7 on naive CD8br cells mediates the effect of *ZDHHC20* on schizophrenia, with a mediation effect of 0.0634, accounting for 33.35% of the total effect (**Figure** **5**; details in Supplementary Table S15).

## 4 Discussion

### 4.1 Key Findings and Their Implications

Schizophrenia remains a formidable challenge in psychiatric research, characterized by its complex etiology and substantial global burden. Using MR, this study provides compelling evidence for a novel causal pathway implicating ZDHHC20, a palmitoyltransferase, in schizophrenia pathogenesis. Our findings delineate a tripartite mechanism:

- *ZDHHC20* Expression and Schizophrenia Risk: Elevated *ZDHHC20* expression is causally linked to increased schizophrenia susceptibility, as demonstrated by robust MR associations (*p* < 0.05, validated by SMR).
- Epigenetic Regulation via DNA Methylation: The CpG site cg18095732 exerts upstream control over *ZDHHC20* expression, mediating 59.31% of its effect on schizophrenia risk (*p* < 0.05). This establishes DNA methylation as a pivotal regulator of palmitoylation in psychiatric disorders.
- Immune Mediation through CCR7: *ZDHHC20* partially influences schizophrenia risk via the immune system, modulating CCR7 expression on naive CD8^+ T cells, with a mediated effect of 33.35% (*p* < 0.05).

These results integrate epigenetic modifications, protein palmitoylation, and immune dysregulation into a cohesive framework, offering a paradigm shift in understanding schizophrenia's molecular basis. Crucially, ZDHHC20 emerges as a promising therapeutic target, bridging neuronal and immune contributions to the disease.

### 4.2 *ZDHHC20* and Palmitoylation in Schizophrenia

Palmitoylation, a reversible post‐translational modification, regulates protein localization, stability, and interactions, profoundly influencing neuronal and immune functions. Within the ZDHHC family of palmitoyltransferases, *ZDHHC20* stands out, expressed in both neurons and immune cells. Previous studies have tied palmitoylation to synaptic plasticity, with aberrant modification of proteins like PSD‐95 and SNAP‐25 linked to neurotransmitter dysregulation in schizophrenia (Linder and Deschenes 2007). Our research extends this narrative, showing that *ZDHHC20*‐mediated palmitoylation not only sustains synaptic integrity but also modulates immune signaling—a dual role distinguishing it from homologs like *ZDHHC5* or *ZDHHC8* (Liao et al. 2023).

In cancer and inflammatory contexts, *ZDHHC20* enhances NLRP3 inflammasome activation via palmitoylation, amplifying immune responses (Zheng et al. 2023). Similarly, our data suggest that *ZDHHC20* may amplify inflammatory cascades through CCR7‐mediated T‐cell activation, exacerbating schizophrenia risk. This aligns with emerging evidence of peripheral immune dysregulation in schizophrenia, where chronic inflammation may disrupt brain‐immune crosstalk, worsening neurodevelopmental and degenerative processes (Du et al. 2024). The specificity of *ZDHHC20*’s immune role merits further investigation, particularly relative to other ZDHHC enzymes, to clarify its unique contribution to schizophrenia pathology.

### 4.3 DNA Methylation as a Regulatory Nexus

DNA methylation, a cornerstone of epigenetic regulation, orchestrates gene expression in response to genetic and environmental cues, with established relevance to psychiatric disorders (Jaffe et al. 2016). We identify cg18095732 as a methylation site regulating ZDHHC20 expression, providing a mechanistic link between epigenetics and palmitoylation in schizophrenia. This finding echoes genome‐wide methylation studies showing altered patterns in the brains and peripheral tissues of schizophrenia patients, impacting genes tied to synaptic plasticity and inflammation (Starnawska and Demontis 2021).

Uniquely, our MR approach establishes causality, revealing that DNA methylation at cg18095732 accounts for over half of *ZDHHC20*’s effect on schizophrenia risk. This suggests that environmental stressors—known to shape methylation profiles (e.g., prenatal infection, chronic stress) (Vidrascu et al. 2019)—may converge on *ZDHHC20* to heighten disease susceptibility. By integrating multi‐omics data, we provide the first causal evidence for an epigenetic‐palmitoylation‐immune axis, positioning DNA methylation as a potential biomarker and therapeutic target in schizophrenia.

### 4.4 Immune Dysregulation and Schizophrenia Pathogenesis

The immune hypothesis of schizophrenia posits that aberrant brain‐immune interactions underpin its etiology, supported by evidence of microglial activation, elevated cytokines, and T‐cell anomalies in affected individuals (Dietz et al. 2020). Our findings refine this model, demonstrating that *ZDHHC20* regulates CCR7 on naive CD8^+ T cells, a chemokine receptor critical for lymphocyte trafficking and neuroimmune communication (Reina‐Campos et al. 2021). This immune mediation suggests that *ZDHHC20*’s palmitoylation activity extends beyond synaptic proteins to immune modulators, potentially amplifying neuroinflammation and disrupting synaptic pruning—a hallmark of schizophrenia.

The partial mediation by CCR7 (33.35%) indicates that additional immune pathways, such as innate immunity or cytokine signaling, may contribute to *ZDHHC20*’s effects. This complexity highlights the need for integrative approaches to dissecting immunity's role in schizophrenia, especially the interplay between peripheral and central immune responses. These insights pave the way for immune‐targeted therapies, where *ZDHHC20* inhibition could mitigate both neuronal and inflammatory drivers of the disease.

### 4.5 Strengths, Limitations, and Future Directions

Strengths:Robust causal inference: MR minimizes confounding and reverse causation, enhancing the reliability of our findings. Multi‐Omics integration: Synthesizing eQTL, pQTL, and mQTL data offers a holistic view of the DNA methylation‐*ZDHHC20*‐immune axis. Therapeutic Potential: Identifying *ZDHHC20* as a nexus of schizophrenia pathology provides a tangible target for precision medicine.

Limitations:MR assumptions: Sensitivity analyses (e.g., MR‐Egger, MR‐PRESSO) address pleiotropy, but unmeasured horizontal effects could influence results. Population Specificity: Reliance on European GWAS data limits generalizability; replication in diverse cohorts is essential. Mechanistic Gaps: Lack of functional validation leaves ZDHHC20's precise molecular interactions unresolved. Although our MR and mediation analyses support a putative causal pathway involving DNA methylation, ZDHHC20, and immune regulation, we did not perform functional validation in cellular, animal, or clinical models. As a result, the mechanistic interpretation remains hypothetical and should be confirmed by future experimental studies.

Future directions:Future studies should leverage single‐cell transcriptomics and CRISPR‐based gene editing to dissect *ZDHHC20*’s cell‐specific roles in neurons and immune cells. Longitudinal epigenome‐wide association studies could further elucidate how dynamic methylation changes correlate with disease progression, informing early intervention strategies.

## 5 Conclusion

This study unveils a novel causal pathway in schizophrenia, wherein DNA methylation at cg18095732 regulates *ZDHHC20* expression, which in turn influences schizophrenia risk through CCR7‐mediated immune modulation. By integrating epigenetic, palmitoylation, and immune mechanisms, we offer a multidimensional perspective on schizophrenia etiology, reinforcing its neuroimmune basis. Identifying *ZDHHC20* as a pivotal mediator underscores its potential as a therapeutic target for epigenetic and immune‐based interventions. These findings herald a new era of precision psychiatry, where multi‐omics approaches illuminate complex disease mechanisms. Future research leveraging advanced functional genomics is critical to translate these insights into clinical advancements, ultimately alleviating the burden of schizophrenia.

## Author Contributions

**Qinghua Guo**: conceptualization, investigation, funding acquisition, writing – original draft, writing – review and editing, visualization, validation, methodology, software, formal analysis, project administration, resources, supervision, and data curation. **Chao Chen**: conceptualization and methodology. **Yong Wang**: conceptualization and methodology. **Libo Guo**: conceptualization and investigation. **Shaomei Shang**: conceptualization, investigation, and funding acquisition.

## Conflicts of Interest

The authors declare no conflicts of interest.

## Peer Review

The peer review history for this article is available at https://publons.com/publon/10.1002/brb3.70722.

## Data Availability

Data used in this study were obtained from deCODE GENETICS (https://www.decode.com/summarydata/), GoDMC (http://mqtldb.godmc.org.uk/downloads), FinnGen data (https://storage.googleapis.com/finngen‐public‐data‐r11/summary_stats/finngen_R11_F5_SCHZPHR.gz), and GWAS data from the Francesco Cucca Study of Immune Cells (https://doi.org/10.1038/s41588‐020‐0684‐4). These databases provide high‐quality, large‐scale datasets that have been widely used in genetics, epidemiology, and clinical research. The authors express their gratitude to the researchers and institutions responsible for curating and maintaining these invaluable resources, which have greatly contributed to the understanding of science and driven impactful analyses.
